# Trimethyl Chitosan/Siloxane-Hybrid Coated Fe_3_O_4_ Nanoparticles for the Uptake of Sulfamethoxazole from Water

**DOI:** 10.3390/molecules24101958

**Published:** 2019-05-21

**Authors:** Sofia F. Soares, Tiago Fernandes, Tito Trindade, Ana L. Daniel-da-Silva

**Affiliations:** CICECO-Aveiro Institute of Materials, Department of Chemistry, University of Aveiro, 3810-193 Aveiro, Portugal; sofiafsoares@ua.pt (S.F.S.); jtfernandes@ua.pt (T.F.); tito@ua.pt (T.T.)

**Keywords:** trimethyl chitosan, magnetic nanoparticles, core@shell, hybrid coating, water treatment, adsorption, sulfamethoxazole

## Abstract

The presence of several organic contaminants in the environment and aquatic compartments has been a matter of great concern in the recent years. To tackle this problem, new sustainable and cost-effective technologies are needed. Herein we describe magnetic biosorbents prepared from trimethyl chitosan (TMC), which is a quaternary chitosan scarcely studied for environmental applications. Core@shell particles comprising a core of magnetite (Fe_3_O_4_) coated with TMC/siloxane hybrid shells (Fe_3_O_4_@SiO_2_/SiTMC) were successfully prepared using a simple one-step coating procedure. Adsorption tests were conducted to investigate the potential of the coated particles for the magnetically assisted removal of the antibiotic sulfamethoxazole (SMX) from aqueous solutions. It was found that TMC-based particles provide higher SMX adsorption capacity than the counterparts prepared using pristine chitosan. Therefore, the type of chemical modification introduced in the chitosan type precursors used in the surface coatings has a dominant effect on the sorption efficiency of the respective final magnetic nanosorbents.

## 1. Introduction

Emerging pollutants encompass a vast number of organic compounds such as pharmaceuticals that, in recent years, have accumulated in aquatic compartments due to continuous and uncontrolled discharge of such substances into the environment [1,2]. The antibiotic sulfamethoxazole is among the pollutants detected in water sources [1,3,4]. Sulfamethoxazole (SMX) is a broad-spectrum antibiotic for human and veterinary use, that belongs to sulfonamide group and that has been identified as a persistent pollutant [5]. Based on its consumption, discharge, persistence and toxic properties, SMX has been considered by several scientists as an antibiotic of particular concern for aquatic environments [4]. 

Most wastewater treatment plants (WWTPs) are not efficient in the removal of emerging pollutants [6,7,8]. The median SMX removal rate in WWTPs worldwide, considering 190 removal rates reported, is about 49% [9]. Given the limitations of conventional treatments used in WWTPs, novel, sustainable and effective technologies are in high demand. Several methods have been proposed for the removal of SMX from water [10], including advanced oxidation processes [11], biological treatment [12], membrane separation [13] and adsorption [14]. Among these methods, adsorption is very attractive in view of its simplicity of implementation, cost-effectiveness, and less production of toxic intermediates. However, so as to achieve highly effective adsorptive separation, sorbent materials with high capacity, chemical selectivity and fast rate of adsorption must be used. This is a challenge and it has boosted the development of new sorbents. Several materials have been proposed for the uptake of sulfamethoxazole from contaminated water through adsorptive methods [15,16,17,18,19]. These sorbents present several drawbacks including low adsorption capacity [15,17], the use of hazardous compounds for aquatic life [20,21], the need of expensive components [18,19] and elaborated synthetic procedures with several steps [19]. Thus, novel sorbent materials are needed. Magnetic nanosorbents with core-shell structure are amongst the most interesting alternatives given the concomitant large surface area and magnetic features that allow for magnetically assisted separation technologies. Magnetic separation can be done using low magnetic fields [22] providing an attractive and cost-effective method for practical operation. Magnetic iron oxides such as magnetite (Fe_3_O_4_) are suitable as core material owing to convenient magnetic properties, low toxicity and price, and facile preparation [23,24,25,26]. Coating of Fe_3_O_4_ is desirable to prevent its oxidation and consequent iron ions leaching and reduction of magnetization. Bare magnetite particles show modest adsorption towards sulfonamide antibiotics [27]. Thus, coating of Fe_3_O_4_ is necessary to provide specific functionalities that can be selective for pollutants uptake and enhance sorption performance. The composition of the coating determines the sorbents surface chemistry characteristics, such as acidity/basicity and charge, that could impose decisive influence on the sorption capacity and mechanism. The search for eco-friendly and low-cost sorbents has prompted the interest for new biopolymer-based nanomaterials and their use in water decontamination [28]. In this context, our group has explored the promising ability of several magnetic biopolymer-siloxane hybrid nanomaterials in the uptake of a wide variety of pollutants from water [29,30,31,32]. Chitosan is among the biopolymers of interest for water treatment purposes owing to its low-cost, biodegradability, low toxicity and chemical functionality [33]. However unmodified chitosan shows little adsorption of SMX and appropriate chitosan modification is required to achieve high adsorption capacity. With this end in view, chitosan modified with immobilized metal cations has been reported for the adsorption of SMX [34] or to enhance the interaction with other sulfonamide antibiotics [35].

N,N,N-trimethylchitosan (TMC) is a quaternary derivative of the polysaccharide chitosan that has received considerable attention in biomedical applications, namely as absorption enhancer in drug delivery [36,37]. TMC, as most of chitosans and derivatives, present low toxicity [38]. In TMC a positive charge is introduced by quaternization of amino group of chitosan. Owing to cationic groups, TMC provides an opportunity for development of novel sorbent compositions with ability to interact with negatively charged pollutants that are present at common pH values found in real waters. This is a clear advantage when compared with unmodified chitosan that displays positive charge only in acidic conditions. Furthermore, TMC shows enhanced antimicrobial activity than original chitosan [39]. Nevertheless, to the best of our knowledge, TMC was barely explored for the development of sorbents for environmental applications and the number of studies reported in this field is scarce [40,41,42].

In this work we have investigated the coating of magnetite nanoparticles with TMC-siloxane hybrid materials. Furthermore, we have explored the potential application of the resulting core@shell type nanoparticles as magnetic nanosorbents for the removal of SMX from water through batch adsorption tests. 

## 2. Materials and Methods

### 2.1. Materials

Chitosan (from shrimp shells, deacetylation degree ≥75%), tetraethyl orthosilicate (Si(OC_2_H_5_)_4_, TEOS, >99%), potassium nitrate (KNO_3_, >99%) and 3-(triethoxysilyl)propyl isocyanate ((C_2_H_5_O)_3_Si(CH_2_)_3_NCO, ICPTES, 95%) were purchased from Sigma–Aldrich (Steinheim, Germany). Trimethyl chitosan was obtained from Henan Tianfu Chemical Co., Ltd (Zhengzhou, China). Potassium hydroxide (KOH, >86%) was purchased from PronoLab (Lisbon, Portugal). Sulfamethoxazole (C_10_H_11_N_3_O_3_S) was obtained from Fluka Chemie (Buchs, Switzerland). Ethanol (CH_3_CH_2_OH, >99%) and ferrous sulfate heptahydrate (FeSO_4_.7H_2_O, >99%) were obtained from Panreac (Barcelona, Spain). *N*,*N*–Dimethylformamide (HCON(CH_3_)_2_) was obtained from Carlo Erba Reagents (Peypin, France). Ammonia solution (25% NH_3_) was purchased from Riedel-de-Häen (Hanover, Germany) and methanol (CH_3_OH, >99%) was purchased from VWR International (Radnor, PA, USA). Milli-Q water was obtained from the Synergy equipment from Millipore with a 0.22 μm filter (Darmstadt, Germany). All chemicals were used without any further treatment. 

### 2.2. Synthesis of the Magnetic Core

The magnetic core (magnetite–Fe_3_O_4_) was prepared by oxidative hydrolysis of iron(II) sulphate heptahydrate (FeSO_4_·7H_2_O) in alkaline medium, under a N_2_ stream, as reported in the literature [43]. First, milli-Q water was deoxygenated with N_2_ under vigorous stirring for two hours. Then, 25 mL of deoxygenated water was added to 250 mL round flask and 1.90 g and 1.52 g of KOH and KNO_3_, respectively, were added. The mixture was heated at 60 °C with bubbling N_2_ and mechanically stirred at 500 rpm. After salt dissolution, 25 mL of an aqueous solution containing 4.75 g of FeSO_4_·7H_2_O was added drop-by-drop and the stirring was increased to 700 rpm. The solution was left to react for 30 min. Then, the round flask was transferred to a hot oil bath (90 °C) and left with no stirring for four hours, under N_2_. The resulting black powder was washed several times with deoxygenated water and ethanol. Finally, the particles were dried at room temperature. 

### 2.3. Coating of the Magnetic Nanoparticles

The coating of the magnetic cores was performed using two distinct chitosans: trimethyl chitosan (TMC) and pristine chitosan (CHIT). The coating was performed using a sol-gel method, and consisted in the hydrolytic co-condensation of a mixture of TEOS with a precursor (SiTMC or SiCHIT) comprising the biopolymer chemically modified with alkoxysilyl groups, carried on in the presence of the magnetic nanoparticles. Briefly, these precursors were generated by reaction between the polysaccharide (1 g) TMC or CHIT, dry dimethylformamide (13 mL), and the silane coupling agent ICPTES (1.3 mL) [30,32,44]. The reaction was performed under an inert atmosphere of dry nitrogen (N_2_), at 100 °C (373 K), and left under constant stirring for 24 h. After cooling at room temperature, the precursors were washed with dry ethanol and dry methanol. Finally, the volatiles were removed under a dynamic vacuum to yield a solid product. For the coatings, a suspension of Fe_3_O_4_ nanoparticles (40 mg) in 38 mL of ethanol was prepared and kept immersed in an ice bath, under sonication (horn Sonics, Vibracell, Newtown, CT, USA). After 15 min, the ammonia (2.4 mL) and a mixture of TEOS (0.406 mL) and precursor (0.4 g) were slowly added to the solution, that was left for 2 h immersed in an ice bath, under sonication. The resulting particles were collected magnetically using a NdFeB magnet, and washed thoroughly with ethanol. Finally, the particles were left to dry by solvent evaporation and two distinct chitosan coated magnetic particles were obtained (Fe_3_O_4_@SiO_2_/SiTMC and Fe_3_O_4_@SiO_2_/SiCHIT). 

### 2.4. Characterization of the Materials

^1^H-NMR spectra of chitosans and derivatives were recorded on Bruker AMX 300 spectrometer at 300.13 MHz (Bruker, Wissembourg, France). Deuterium oxide (D_2_O), deuterium chloride (DCl) and deuterated chloroform (CDCl_3_) were used as solvents, the chemical shifts are expressed in δ (ppm) and the coupling constants (*J*) in hertz [Hz]. The morphology and size of the particles was analyzed by transmission electron microscopy (TEM), using a Hitachi H-9000 TEM microscope (Chiyoda, Tokyo, Japan) operating at 300 kV. Samples for TEM analysis were prepared by evaporating the diluted suspensions of the nanoparticles on a copper grid coated with an amorphous carbon film (Agar Scientific, Stansted, Essex, UK). The particle diameter and the thickness of the siliceous shell were measured by analysis of the electron micrographs using ImageJ software (version 1.8.0, https://imagej.nih.gov, National Institutes of Health–NIH, Bethesda, Maryland, MD, USA). At least 50 nanoparticles were measured for each sample. Fourier transform infrared (FTIR) spectra of the particles were measured in solid state and the spectra of the materials were collected using a Bruker Optics Tensor 27 spectrometer (Billerica, MA, USA) coupled to a horizontal attenuated total reflectance (ATR) cell, using 256 scans at a resolution of 4 cm^−1^. The specific surface area of the particles was assessed by nitrogen adsorption Brunauer–Emmett–Teller (BET) measurements performed with a Gemini V2.0 surface analyzer (Micromeritics Instrument Corp. Norcross, GA, USA) at −196 °C. Prior to BET measurements, the samples were degassed at 80 °C under nitrogen flow overnight. Elemental analysis of carbon, nitrogen and hydrogen was obtained on a Leco Truspec-Micro CHNS 630-200-200 (Saint Joseph, MI, USA). Thermogravimetric analysis (TGA) was performed using a TGA 50 Shimadzu equipment (Shimadzu, Columbia, MD, USA). Samples were heated from 25 °C to 900 °C at 10 °C/min, in air. The surface charge of the nanoparticles was given by zeta potential that was determined through electrophoretic light scattering performed in aqueous solutions of the particles, in a Zetasizer Nano ZS equipment from Malvern Instruments (Malvern, UK). A Perkin Elmer Analyst 100 apparatus (Analytical Instrument Resource, Golden, CO, USA) was employed for the iron quantification in solution using Atomic Absorption spectrophotometry (AAS). Sulfamethoxazole concentrations were determined spectrophotometrically by monitoring the absorbance at 265 nm using quartz cells in a GBC Cintra-303 UV-Vis spectrophotometer (GBC Scientific Equipment, Hampshire, IL, USA) and deionized water as reference.

### 2.5. Uptake of Sulfamethoxazole from Aqueous Solutions

Batch adsorption tests were performed to investigate the suitability of the coated magnetic particles (Fe_3_O_4_@SiO_2_/SiTMC and Fe_3_O_4_@SiO_2_/SiCHIT) for the uptake of sulfamethoxazole (SMX) from water. SMX stock solutions were prepared by dissolving an appropriate amount of the compound in ultra-pure water and stirring overnight in dark conditions. Solutions with initial SMX concentration of 40 and 80 mg/L were prepared by diluting the stock SMX solution, and the experiments were conducted at an initial pH of 5. For adsorption experiments performed at distinct pH values (4, 5, 6, 7, 8 and 9), solutions of NaOH (0.1 M) and HCl (0.1 M) were used for pH adjustment. The effect of these pH values was tested at an initial SMX concentration of 40 mg/L. The aqueous solutions of SMX were freshly prepared before each experiment.

Batch experiments were carried out by adding precisely weighted amounts of each coated magnetic particles to a SMX aqueous solution of known concentrations in glass vials and shaken using a vertical rotator at a constant rotation speed (30 rpm), under isothermal conditions (25 ± 1 °C), for 24 h. The sorbent dosage tested was 0.5 mg/mL in all the experiments. Aliquots were collected for analysis at different times, and the magnetic nanosorbents were separated magnetically using a NdFeB magnet. The removal efficiency of SMX was assessed by measuring the amount of antibiotic that remained in solution after being in contact with the coated magnetic particles. Sulfamethoxazole concentration in the supernatant was determined spectrophotometrically by monitoring the absorbance at 265 nm, respectively, in a UV-Vis spectrophotometer (GBC Scientific Equipment, Hampshire, IL, USA). Plotting the absorbance against SMX concentration, the calibration curve was given by the best data fit by a linear least square equation (Figure S1, Supporting Information), that was used to convert the absorbance into SMX concentration, for all analyzed samples.

The amount of SMX adsorbed per mass unit of particles, at time *t*, (q_t_ in mg/g) was estimated from the mass balance between its initial concentration (C_0_ in mg/L) and the concentration at time *t* (C_t_ in mg/L) in solution, as displayed by Equation (1), where *V* (L) is the total volume of SMX solution and *m*, expressed in g*,* is the dry weight of the magnetic adsorbents:

(1) $$q_{t}=\left(C_{0}-C_{t}\right)\times \frac{V}{m\;}\;,$$

The removal capacity (R, expressed in %) was calculated from Equation (2):

(2) $$R=\frac{C_{0}-C_{t}}{C_{0}}\times 100,$$

Control uptake experiments, i.e., in the absence of sorbent particles, were also carried out in parallel under the same conditions to confirm if SMX losses were negligible. Equilibrium isotherms for the removal of SMX from aqueous solutions were obtained by using SMX solutions with different concentrations (20, 40, 70, 90, 140, 160, 180 and 195 mg/L), at pH 5 and 25 °C for 24 h. The amount of SMX adsorbed at equilibrium (q_e_, mg/g) was assessed by UV–Vis spectroscopy (GBC Scientific Equipment, Hampshire, IL, USA) and calculated using Equation (1) for C_t_ = C_e_, where *C_e_* (mg/L) is the concentration of the solute at equilibrium. To assess the stability of Fe_3_O_4_@SiO_2_/SiTMC and Fe_3_O_4_@SiO_2_/SiCHIT nanoparticles in aqueous medium, 2.5 mg of particles were dispersed in 5 mL of distilled water at pH = 5 and left stirring for 8 h. Afterwards the particles were separated magnetically and the supernatant was analyzed using AAS for Fe content. A Perkin Elmer Analyst 100 apparatus was employed for the iron quantification.

In order to investigate the reusability of the sorbent particles, 10 mg of magnetic particles were loaded with SMX using 20 mL of an aqueous solution at a concentration of 80 mg/L. For desorption, the SMX-loaded particles were collected, rinsed three times with 20 mL ethanol, magnetically separated and dried. After SMX desorption, the particles were reused in adsorption experiments and the process was repeated for four times.

## 3. Results and Discussion

### 3.1. Characterization of Chitosan Polymers

**Scheme 1:**
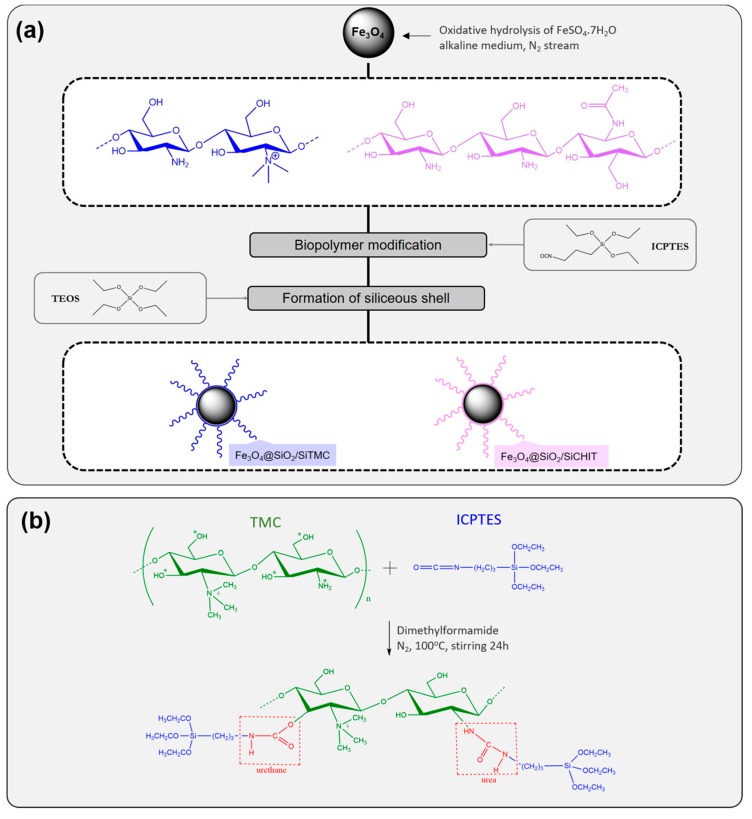
Scheme illustrating (**a**) the coating of Fe_3_O_4_ nanoparticles with siliceous shells enriched in biopolymers; (**b**) chemical route to obtain an alkoxysilane derivative of trimethyl chitosan (TMC) (SiTMC) from reaction between TMC and ICPTES.

In this work, magnetic Fe_3_O_4_ nanoparticles have been coated with hybrid siliceous shells enriched in trimethyl chitosan (TMC) which is a quaternary chitosan of interest for biomedical and environmental applications. The coating process followed a one-step procedure, comprising the hydrolysis and condensation of a mixture tetraethyl orthosilicate (TEOS) and an alkoxysilane derivative of TMC, carried on in the presence of the magnetic particles (Scheme 1).

**Figure 1:**
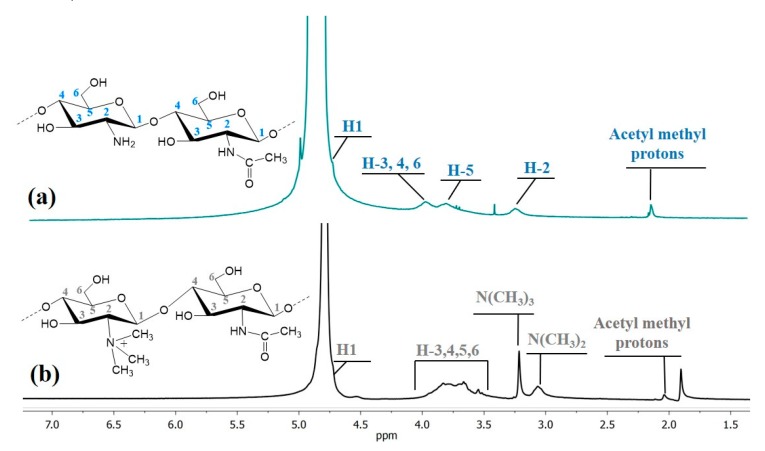
^1^H-NMR spectra of (**a**) pristine chitosan (in DCl); (**b**) TMC (in D_2_O).

As previously reported, this synthetic approach allows the coating of Fe_3_O_4_ particles with siliceous shells enriched with neutral and anionic polysaccharides [31,32]. More recently, we succeeded in applying this methodology to the coating of magnetic nanoparticles with chitosan which is a cationic polysaccharide [44]. Herein, besides TMC, pristine chitosan (CHIT) was also used for comparison effects. These two polysaccharides have similar backbone structure but differ in chemical functionality as evidenced by ^1^H-NMR spectroscopy results (Figure 1). The spectrum of chitosan (Figure 1a) shows the proton resonances of the glycopyranose unit [45]: H1 at 4.8 ppm, H2 at 3.2 ppm and H3-H6 at 4.0–3.5 ppm. The protons from acetyl group appear at 2.4 ppm. Overall, these resonances were visible in the spectrum of TMC. In the spectrum of TMC (Figure 1b) a new resonance appeared at 3.4 ppm that is ascribed to the methyl group at the *N*,*N*,*N*-trimethylated site [46,47,48,49]. The resonance at 3.1 ppm suggests also dimethylation of TMC [50] that was further confirmed by the appearance of a new resonance at 46.8 ppm in the ^13^C-NMR spectrum (data not shown). The degree of quaternization calculated from ^1^H-NMR data (Equation (S1), Supporting Information) was 15.3% for TMC.

The alkoxysilane derivative of TMC (SiTMC), was prepared by reacting an organosilane with isocyanate functionality (ICPTES) with the polymer TMC (Scheme 1b). The isocyanate groups can react with hydrogen labile groups, such as amine and hydroxyl groups, present in TMC. Note that the degree of quaternization of TMC was 15.3%, indicating the presence of primary amines in the structure of TMC that could react with isocyanate to form urea type covalent bonds. In the ^1^H-NMR spectrum of SiTMC (Figure S2, Supporting Information) novel resonances could be ascribed to –NH– in urethane (5.3 ppm in CDCl_3_) and –CH_3_ from etoxy groups (1.0 ppm in CDCl3) that confirm the reaction between TMC and ICPTES [51].

### 3.2. Characterization of Fe_3_O_4_ Coated Particles

**Figure 2:**
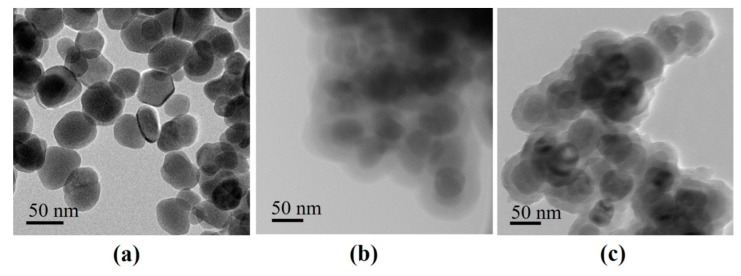
TEM images of neat and surface modified nanoparticles: (**a**) Fe_3_O_4_; (**b**) Fe_3_O_4_@SiO_2_/SiTMC and (**c**) Fe_3_O_4_@SiO_2_/SiCHIT.

Magnetite nanoparticles (Fe_3_O_4_) were synthesized by oxidative hydrolysis of Fe(II) in alkaline conditions [43]. The synthesis yielded a black precipitate that could be isolated by magnetic separation. The powder X-ray diffraction (XRD) patterns of the prepared particles (Figure S3, Supporting Information) matched well the diffraction patterns reported for magnetite (JCPDS file No. 19-0629, space group Fd3m) [52], and confirm that Fe_3_O_4_ is the main crystalline phase present in its composition. The transmission electron microscopy (TEM) images indicate that Fe_3_O_4_ particles were spheroidal in shape with an average size of 54 ± 9 nm (Figure 2a and Figure S4, Supporting Information). After hydrolysis and condensation of a mixture of alkoxysilane derivative of the biopolymer with TEOS, performed in the presence of Fe_3_O_4_ particles, the particles appear uniformly coated with amorphous shells (Figure 2b,c). The thickness of the coatings, determined from TEM images, were 26 ± 3 nm for TMC particles and 14 ± 2 nm for CHIT particles, respectively. It is clear that this approach yields thicker coatings when quaternary chitosan is involved, in comparison with pristine chitosan. The coated particles were magnetic and were quickly separated from the solution using a bench magnet (Figure S5, Supporting Information). In our previous studies with identical Fe_3_O_4_ nanoparticles, we have confirmed that these are ferrimagnetic with a saturation magnetization and coercivity values of 84 emu/g and 100 Oe, respectively [43]. In coated particles the magnetization saturation is expected to decrease owing to the diamagnetic siliceous shell [43]. Nevertheless, the coated particles still exhibit magnetic characteristics that make them suitable for magnetically assisted separation.

The specific surface area (S_BET_) and the total porosity of the particles (V_p_) was assessed by N_2_ sorption/desorption technique. The coated particles have a similar specific surface area of 7 m^2^/g (Table 1) and low total porosity (<0.01 cm^3^/g), indicating that the coating has no relevant porosity. The BET specific surface areas (S_BET_) of the bare magnetite and coated particles, were compared with the theoretical specific surface areas (S) making use of the S = 6/(D × ρ) relationship, where ρ is particle density. This parameter was taken as 5.20 g/cm^3^, for bare magnetite. For coated magnetites it was assumed that the density of the coating was the density of amorphous SiO_2_ (2.2 g/cm^3^) and the theoretical surface area was estimated from Equation (S2) (Table S1, Supporting Information). Coated magnetite shows similar S values than bare magnetite. This is explained by a combined influence of the increase of particle diameter and decrease of particle density, these parameters having opposite effects on the resulting specific surface area. The BET specific surface areas were of the same order of magnitude than the geometric specific surface area values but consistently lower. This difference was more marked in coated particles and might suggest to some extent the formation of coated particles containing several cores inside. Nevertheless, multicore particles were not detected in TEM micrographs.

**Figure 3:**
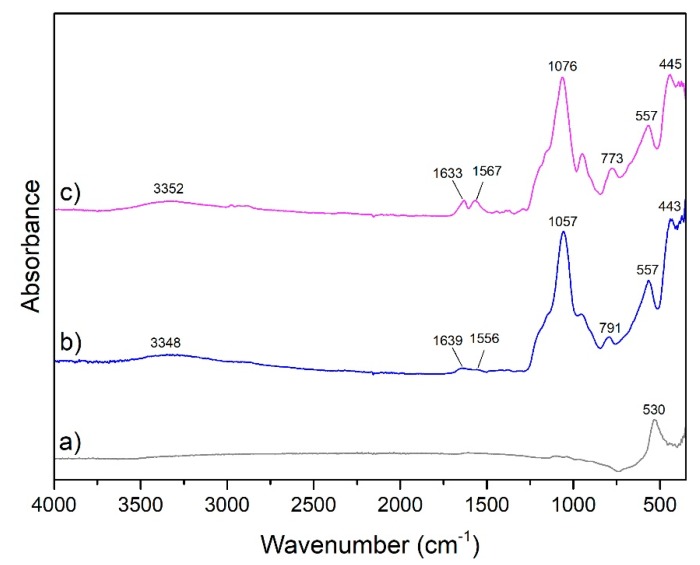
FTIR-ATR spectra of the nanoparticles: (a) Fe_3_O_4_; (b) Fe_3_O_4_@SiO_2_/SiTMC and (c) Fe_3_O_4_@SiO_2_/SiCHIT.

FTIR spectroscopy of the coated materials was performed to assess the composition of the particles’ coating. The spectrum of magnetite (Figure 3a) shows a strong band centered around 530 cm^−1^ that is ascribed to the Fe–O stretching vibration in the Fe_3_O_4_ lattice [43]. A similar band is also visible in the spectra of the subsequent coated particles, with a shift of around 27 cm^−1^ to higher wavenumbers. The bands centered in the range 770–790 cm^−1^ and 443 cm^−1^ can be ascribed to symmetric Si–O–Si stretching and O–Si–O deformation modes of amorphous SiO_2_, respectively [53,54] and indicates the formation of a polysiloxane network in the coatings. The broad and intense band centered at 1057–1076 cm^−1^ results from overlapped vibrational contributions of the silica and the chitosan polymers [44]. Also, the broad band at *ca*. 3350 cm^−1^ can be ascribed to O–H stretching, either from chitosans or silanol groups from silica. In the spectra of the coated particles two new bands appear in the range of 1633–1639 cm^−1^ and 1556–1567 cm^−1^ that could suggest the presence of chitosan polymers. The former can be ascribed to C=O stretching (amide I) in chitosans [55] or to C=O stretching mode in urethane groups [56,57]. The latter is ascribed to C=O stretching in urea groups [56,57]. Both urethane and urea bonds are expected to be formed in the alkoxysilane derivative of the chitosans, used in this coating process, as depicted in Scheme 1. These bands confirm that the chitosan and TMC are covalently linked to siloxane network, which is important to avoid polymer leaching from the coatings. The presence of the chitosans in the coated particles was confirmed by elemental microanalysis (Table 1). Uncoated magnetite shows negligible carbon amount. In contrast, coated particles show important carbon (28 wt%) and nitrogen content (ca. 5 wt%) that is coming from the organic (biopolymer) component (Table S2, Supporting Information). These results confirm that the coatings prepared from TMC and pristine chitosan are enriched in biopolymer, which is in line with previous observations [30]. The biopolymer content estimated from the elemental analysis was 76 wt% and 72 wt%, respectively for TMC and chitosan-based particles.

**Figure 4:**
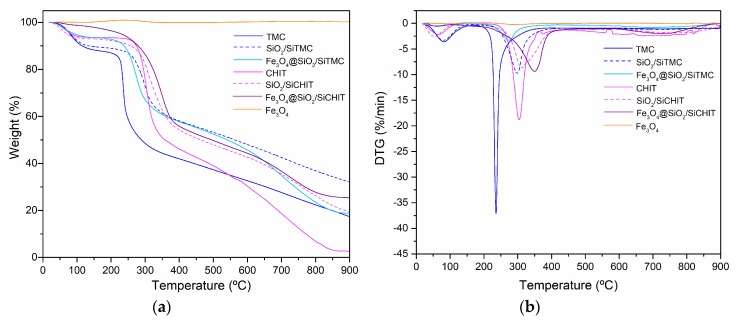
Curves of (**a**) TGA and (**b**) DTG of the biopolymers, siloxane hybrid materials prepared using the biopolymers, bare Fe_3_O_4_ and the coated magnetic hybrid particles.

Further insight into the nature of the coatings was provided by thermogravimetric analysis (TGA) of non-magnetic biopolymer-siloxane hybrid materials of composition identical to the coatings and the coated magnetic hybrid particles. The non-magnetic materials were prepared by employing the chemical route applied in the coating procedure but in this case in the absence of Fe_3_O_4_ nanoparticles. The TGA analysis (Figure 4) provided complementary information regarding the composition and thermal properties of the coatings. The TGA curves of the non-magnetic biopolymer-siloxane hybrid materials exhibit two main stages of weight loss. The first one occurs in the 50–140 °C range due to loss of adsorbed water. Siliceous materials prepared from TMC showed more weight loss at this stage (13–14%) than those prepared from pristine chitosan (6%). Similar trend was observed in the TGA of the original polymers, CHIT and TMC, and of the coated particles, and it is due to enhanced hydrophilicity of the chitosan subjected to quaternization [58]. The second stage of weight loss takes place from 230 to 400 °C and it is due to carbohydrate-backbone fragmentation, including the deacetylation of chitosan and the decomposition of substituted site [59]. This stage begins at lower temperatures for TMC, showing that this quaternary derivative are less thermal stable than the unmodified chitosan [50]. In the siloxane hybrid prepared from TMC this decomposition stage starts at higher temperatures than in the polymer component, thus indicating better thermal stability due to the formation of the polysiloxane network. This is further confirmed by the displacement of the peak temperature in derivative thermogravimetric (DTG) curves to higher temperatures and the decrease of peak area (Figure 4b). Conversely, the hybrid SiO_2_/SiCHIT started to decompose at lower temperature than chitosan (210 °C vs 230 °C). Nevertheless, the maximum decomposition rate temperature is higher in the hybrid material (312 °C vs 303 °C). At 900 °C, the hybrid materials show more residue mass, ca. 15–17 wt.% more than in the corresponding polymer counterpart, which is in agreement with the presence of a siliceous inorganic component in the hybrid material. Regarding magnetic hybrid particles it can be observed that the incorporation of the magnetic core has opposite effect on the thermal stability of TMC and chitosan-based hybrid materials. Thus, the second stage of weight loss starts at lower temperature for Fe_3_O_4_@SiO_2_/SiTMC, and at higher temperature for Fe_3_O_4_@SiO_2_/SiCHIT, when compared to the non-magnetic counterparts. This distinct effect is in agreement with the complexity of the mechanism of polymer thermal decomposition in magnetic nanocomposites, as observed in previous studies. For example, several works report the decrease in the initial decomposition temperature of dextran coated magnetite or in chitosan/magnetite nanocomposites, in comparison with the respective polymer component, an effect that was ascribed to the catalytic action of magnetite on the thermal decomposition of the polymer [60,61]. However, an enhancement of the thermal stability of chitosan and other polymers owing to the incorporation of magnetite nanoparticles has also been described [62,63]. 

**Figure 5:**
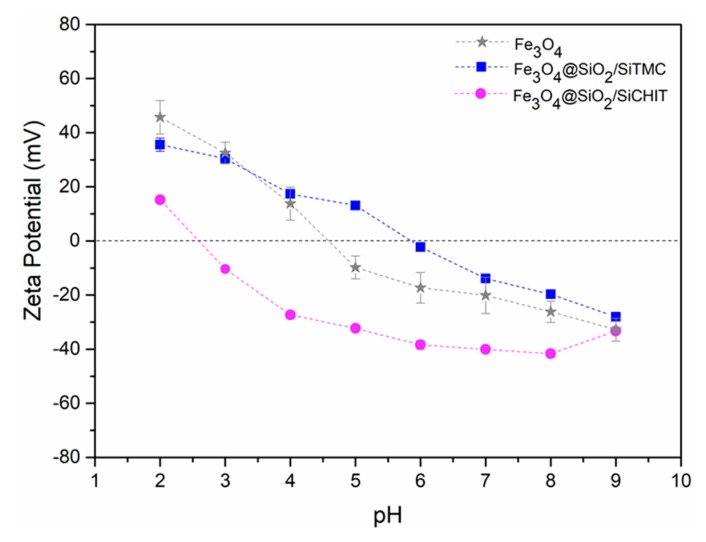
Zeta potential values of bare magnetite and coated particles at variable pH.

The distinct surface composition of the coated particles was further confirmed by differences in the surface charge of the nanoparticles (Figure 5). The surface charge was assessed by zeta potential (ζ) measurements at variable pH. Bare magnetite (Fe_3_O_4_) present an isoelectric point (iep) at ca. 4.5 that is relatively below the usual IEP values reported for magnetite [64] and can indicate oxidation of the particles surface [65]. Nevertheless the IEP value here obtained is in agreement with values reported for bare Fe_3_O_4_ nanoparticles prepared by oxidative hydrolysis [66] and co-precipitation methods [67]. At lower pH values the surface is positively charged while at higher pH values the overall charge of the surface is negative. The isoelectric point shifts to high values in the nanoparticles coated with siliceous shells of TMC (~6). This shift can be ascribed to cationic trimethylammonium groups present on particles surface. In contrast, the particles coated with chitosan siliceous shells show much lower iep (~2.5). This value is similar to IEP of amorphous silica coated particles as observed in our previous studies [44] and is consistent with the reaction of primary amine groups of chitosan with ICPTES to form urea groups.

### 3.3. Uptake of Sulfamethoxazole 

#### 3.1.1. Effect of Initial pH

**Figure 6:**
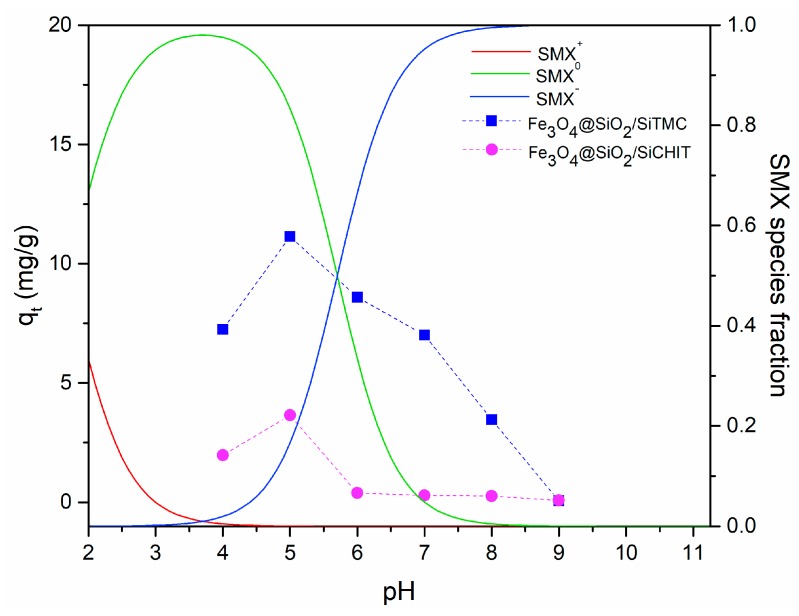
Effect of pH of the medium on the removal of sulfamethoxazole from water, for an initial SMX concentration of 40 mg/L and 24 h contact time and speciation of SMX [68].

The influence of pH on the SMX adsorption was investigated in the range of 4–9 for 24 h contact time, and the results are included in Figure 6. The pH affected the surface charge of the magnetic particles and determined the species of SMX in the solution. Figure 6 shows that a pH 5 enhances the SMX removal efficiency and capacity for TMC-based magnetic particles, and therefore the kinetics and equilibrium studies were carried out at this pH value. Sulfamethoxazole is an amphoteric compound and exists in the environment as cation, neutral molecule and anion, depending on the pH, due to the protonation of the aromatic amine (–NH_3_^+^–, pKa ≈ 1.7) and the deprotonation of sulfonamide group (–SO_2_NH–,pKa ≈ 5.7) [68]. The speciation curves of SMX calculated with the pKa values are depicted in Figure 6. At pH 5, SMX neutral species are dominant. Previous studies demonstrated the relevance of H-bonding interactions between neutral sulfonamide groups and the amine and hydroxyl groups from the sorbents surface in the SMX adsorption [69,70]. These chemical groups are present in chitosan and quaternary derivatives. However, the highest SMX adsorption capacity was achieved using Fe_3_O_4_@SiO_2_/SiTMC particles. This indicates that the sorption mechanism may involve other pathways. Indeed, the NMR analysis of TMC revealed also dimethylated groups (–N–(CH_3_)_2_), that were absent in pristine chitosan. These groups are hydrophobic and may complement SMX adsorption through hydrophobic interactions [71,72]. Nevertheless, because the SMX sorption capacity of chitosan-based particles was much lower, we can assume that quaternary amine groups of TMC play a relevant role in the sorption of SMX. We should note that at pH 5 near 20% of SMX molecules are in the anionic form and thus could interact electrostatically with trimethylammonium groups of the sorbent particles [73]. In alkaline conditions, the SMX adsorption dramatically decreases, most likely owing to electrostatic repulsion between anionic SMX molecules and the surface of the sorbents, that is negatively charged at these pH values.

#### 3.1.2. Kinetic Studies and Effect of Initial SMX Concentration

**Figure 7:**
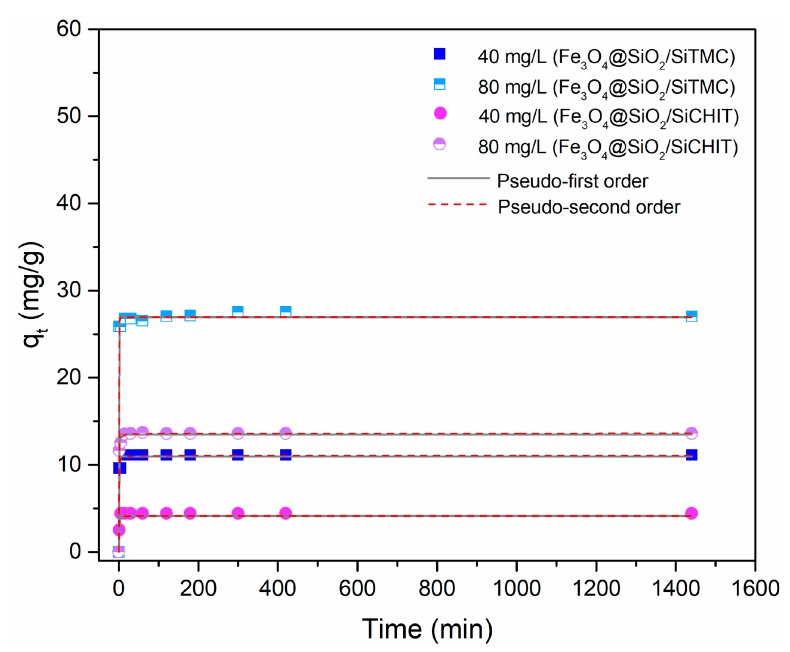
Time profile of adsorption capacity of SMX at variable SMX initial concentration (40 and 80 mg/L) and the corresponding kinetic model fitting using pseudo 1st and pseudo 2nd order equations.

The effects of contact time and initial SMX concentration on the adsorption of SMX by the coated magnetic particles were studied to gain further insight into the adsorption process. Control experiments carried out in parallel in the absence of sorbent particles under the same conditions of pH and contact time have shown negligible losses of SMX (Figure S6, Supporting Information). Figure 7 shows the time profile of SMX uptake using Fe_3_O_4_@SiO_2_/SiTMC and Fe_3_O_4_@SiO_2_/SiCHIT sorbents, for variable initial SMX concentration of 40 and 80 mg/L, at pH=5. The adsorption capacity increased with increasing initial SMX concentration, and for all the cases, a rapid adsorption took place at the beginning (Figure S7, Supporting Information), i.e., for short contact times (ca. 15 min) between the sorbent and the solution. As seen in Figure 7, increasing the initial SMX concentration from 40 to 80 mg/L, the adsorption at equilibrium increases from 11.1 to 27.1 mg/g for Fe_3_O_4_@SiO_2_/SiTMC and 3.6 to 13.6 mg/g for Fe_3_O_4_@SiO_2_/SiCHIT.

Aiming to understand the sorption mechanism between the sorbents and SMX, the time profile data were analyzed using two kinetic models that are commonly used in the study of solid-liquid adsorption processes: the pseudo-first-order equation [74] and the pseudo-second-order equation [75]. The kinetic parameters and the evaluation of the goodness of the fits, obtained by non-linear regression analysis, are reported in Tables S3 and S4 (Supporting Information), and the kinetic fittings are shown in Figure 7. Non-linear form of the kinetic equations was fit to data (Equations (S3) and (S4), Supporting Information), using the curve fitting tool of GraphPad Prism version 7. The goodness of the fit was determined based on the correlation coefficient (R^2^) and the Chi-square test value (χ^2^), (Equations (S5) and (S6), Supporting Information). Both kinetic models described well the experimental data, with R^2^ values ranging from 0.981 to 0.998. This suggests that the adsorption of SMX molecules is the rate-limiting step and that the interaction is of chemical nature. Furthermore, both kinetic models provided good prediction of q_e_ values, (Tables S3 and S4, Supporting Information). 

#### 3.1.3. Equilibrium Isotherm Experiments

**Figure 8:**
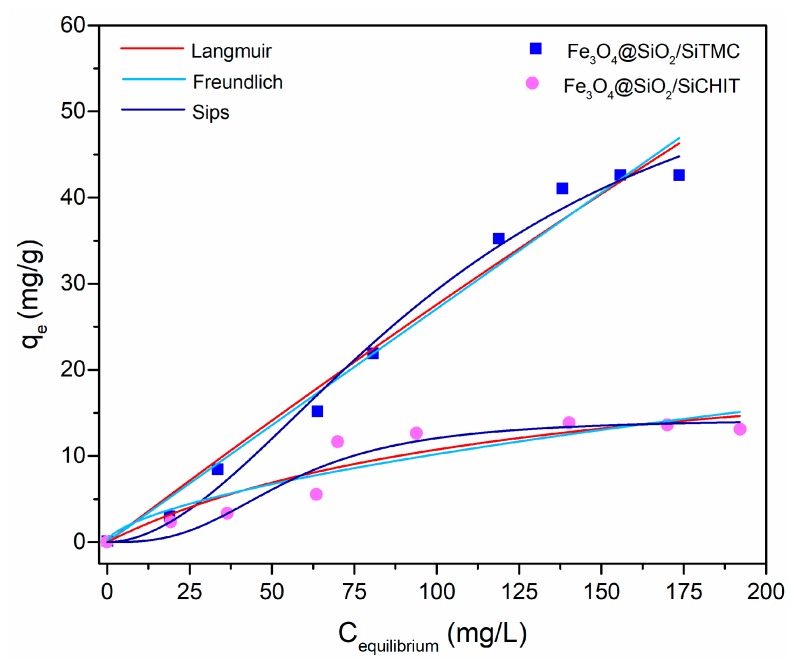
Isotherm data for the adsorption of sulfamethoxazole on Fe_3_O_4_@SiO_2_/SiTMC and Fe_3_O_4_@SiO_2_/SiCHIT particles, and model fitting.

The equilibrium concentration data were obtained from batch adsorption of SMX onto the systems Fe_3_O_4_@SiO_2_/SiTMC and Fe_3_O_4_@SiO_2_/SiCHIT for initial concentrations ranging from 20 to 195 mg/L (Figure 8). To further elucidate the interactions between the sorbents and the sorbate SMX, the sorption data were correlated with Langmuir [76] and Freundlich [77] isotherms models, which are two-parameter isotherms, and Sips [78] isotherm which is a three-parameter isotherm (Equations (S8)–(S10), Supporting Information). The obtained isotherm model parameters are listed in Table 2. Non-linear form of the isotherms equations was fit to data, using the curve fitting tool of GraphPad Prism version 7. The goodness of the fit was determined based on the correlation coefficient (R^2^) and the Chi-square test value (χ^2^), (Equations (S5) and (S6), Supporting Information). Based on fitting indicators, the two-parameter isotherm model that best fits the experimental data for both systems is the Langmuir isotherm. The monolayer adsorption capacity predicted by this model was 597.9 and 24.15 mg/g, for Fe_3_O_4_@SiO_2_/SiTMC and Fe_3_O_4_@SiO_2_/SiCHIT particles, respectively. This model assumes that the adsorption process is most likely to occur by a formation of a monolayer on a homogeneous surface [76,79], instead of the possible multilayer formation in the Freundlich model. Although the Langmuir model provides satisfactory data fitting, this model overestimated the maximum adsorption capacity (q_max_) of the system Fe_3_O_4_@SiO_2_/SiTMC and consequently it is not suitable to predict the experimental data. Actually, the experimental q_max_ value of TMC coated particles was 42 mg/g, while the Langmuir model predicted a value of 598 mg/g. Overall, the Sips isotherm is the model that provides higher R^2^ and lower χ^2^ values, thus being the model that best describes the experimental isotherm data for both systems. The Sips isotherm combines the Langmuir and Freundlich isotherm models. Our results indicate that Fe_3_O_4_@SiO_2_/SiTMC particles adsorb around 42 mg/g of SMX in short contact times, being very effective adsorbents compared to other magnetic sorbents reported in the past [15,17]. Taking into account the economic applicability of the nanosorbents for the removal of SMX, the equilibrium time is one of the most important parameters affecting the design of new adsorbents. The fast adsorption ability suggests that TMC-based magnetic nanosorbent would be a potential adsorbent for the removal of SMX from water. Moreover, these materials are eco-friendly alternatives to sorbents prepared with components considered toxic to aquatic life [21].

#### 3.1.4. Sorbent Stability, Regeneration and Reuse

The TGA curves of the dried particles before and after SMX adsorption were similar, with small differences at the first weight loss stage owing to higher moisture content of the particles after adsorption experiments (Figure S8, Supporting Information). In addition, the residue mass at 900 °C was identical, before and after SMX adsorption. These results indicate that there is no relevant polymer leaching from the particles during the adsorption tests. Elemental microanalysis results (Table S5, Supporting Information) are in agreement with these observations, since after adsorption there was no decrease of the carbon content of the particles. Conversely, a slight increase in the carbon and nitrogen content was observed, that can be ascribed to the adsorbed SMX molecules. To get further insight into the chemical stability of the nanoparticles in aqueous medium, the amount of iron leached from these particles at pH = 5 was quantified using AAS. The amount of Fe leached was below the detection limit, which was 19 μg/L, which indicates that the leaching of Fe ions from the magnetic core is negligible. Hence, the nanoparticles display good chemical stability under the conditions chosen for the SMX uptake experiments, that is provided by the hybrid shell containing biopolymer covalently linked to the siliceous network.

**Figure 9:**
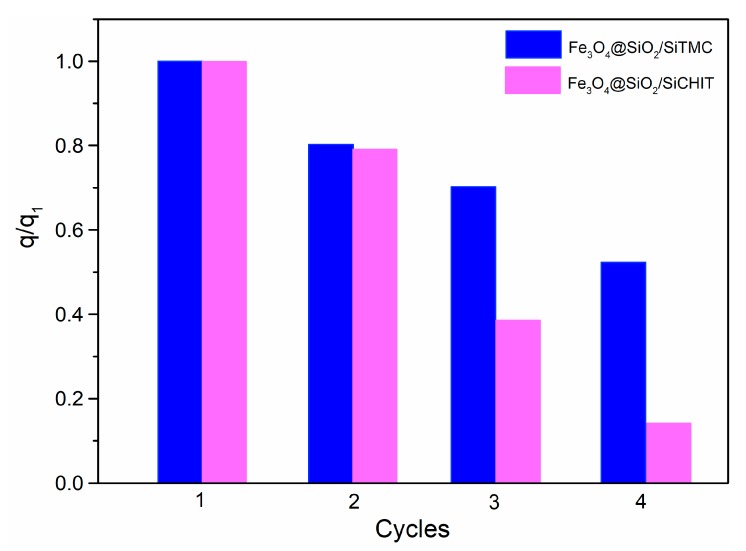
Ratio between the adsorption capacity (q) and the adsorption capacity after first use (q_1_) for Fe_3_O_4_@SiO_2_/SiTMC and Fe_3_O_4_@SiO_2_/SiCHIT particles in consecutive adsorption/desorption cycles.

The ability of Fe_3_O_4_@SiO_2_/SiTMC and Fe_3_O_4_@SiO_2_/SiCHIT to be regenerated and reused in SMX adsorption was assessed by running four consecutive adsorption/desorption cycles. The results presented in Figure 9 show that these sorbents can be reused in the removal of sulfamethoxazole. Nevertheless, overall there is a gradual decrease in SMX adsorption in consecutive cycles that is more marked in chitosan derived particles. For both particles, the adsorption capacity decreased about 20% after the first desorption step. At the 4th cycle the adsorption capacity in TMC particles decreased to half while in CHIT particles a more noticeable decrease to about 15% of the initial adsorption capacity is visible. The results show that TMC based particles can be recycled for SMX adsorption using ethanol, but further efforts must be done to enhance the adsorption capacity in consecutive cycles.

## 4. Conclusions

Magnetite nanoparticles uniformly coated with biopolymer-siloxane hybrid shells of chitosan and its quaternary derivative (TMC) were successfully prepared through a simple single-step coating procedure. The structural and morphological characterization indicated core@shell type morphology, with shells enriched in the biopolymer component.

Among the coated particles prepared, the Fe_3_O_4_@SiO_2_/SiTMC particles showed the greatest performance for the removal of sulfamethoxazole (SMX) from water. The observed adsorptive properties of TMC-based particles can be ascribed to a combined adsorption mechanism that involves electrostatic interactions and hydrophobic interactions between SMX and the trimethylated and dimethylated sites of TMC at sorbents surface. The Fe_3_O_4_@SiO_2_/SiTMC particles have shown a greater adsorption capacity towards SMX when compared to other magnetic sorbents previously reported. These findings demonstrate the potential of Fe_3_O_4_@SiO_2_/SiTMC particles for the uptake of the antibiotic SMX from water, taking advantage of adsorptive and magnetic properties in a single material. Further studies are in progress to assess the full adsorptive performance of these sorbents in real water samples using different operational conditions, and to better understand the sorption mechanism.

## Figures and Tables

**Table 1:** Compositional and morphological properties of uncoated and coated Fe_3_O_4_ nanoparticles.

Sample	C (%) ^1^	H (%) ^1^	N (%) ^1^	D (nm) ^2^	S_BET_ (m^2^/g) ^3^	V_P_ (cm^3^/g)
Fe_3_O_4_	0.06	0.16	0.01	54 ± 9	13.6	0.027
Fe_3_O_4_@SiO_2_/SiTMC	28.6	5.3	5.1	98 ± 11	7.03	0.007
Fe_3_O_4_@SiO_2_/SiCHIT	28.5	5.8	5.3	68 ± 11	7.74	0.007

^1^ Carbon, hydrogen and nitrogen content measured by elemental microanalysis. ^2^ Particle diameter assessed by TEM. ^3^ BET surface area (S_BET_) and porosity volume (V_P_) assessed by N_2_ sorption isotherms.

**Table 2:** Equilibrium model parameters obtained from model fitting to experimental sorption data of Fe_3_O_4_@SiO_2_/SiTMC and Fe_3_O_4_@SiO_2_/SiCHIT, together with the goodness of the fittings.

Isotherm	Model Parameters			Goodness of Fit	
Langmuir	q_L_ (mg/g)	K_L_ (L/mg)		R^2^	χ^2^
Fe_3_O_4_@SiO_2_/SiTMC	598.0	0.0004		0.9796	7.123
Fe_3_O_4_@SiO_2_/SiCHIT	24.1	0.0080		0.8799	4.221
Freundlich	K_F_ (mg^(1−1/*n*)^·L^(1/*n*)^·g^−1^)	*n*		R^2^	χ^2^
Fe_3_O_4_@SiO_2_/SiTMC	0.3582	1.3241		0.9788	7.417
Fe_3_O_4_@SiO_2_/SiCHIT	0.1105	1.247		0.7225	9.759
Sips	q_S_ (mg/g)	Ks (mg/L)^−1/^^β^^S^	β_S_	R^2^	χ^2^
Fe_3_O_4_@SiO_2_/SiTMC	63.1	0.0001	0.5306	0.9911	3.614
Fe_3_O_4_@SiO_2_/SiCHIT	14.32	0.00001	0.3500	0.9259	3.057

## Supplementary Materials

The following are available online: Figure S1: Calibration curve for sulfamethoxazole determination using UV-Vis spectroscopy, Figure S2: ^1^H-NMR spectra of TMC and SiTMC (in CDCl_3_), Figure S3: Powder XRD pattern of magnetite, Figure S4: Histogram of particle diameter of Fe_3_O_4_ nanoparticles, Figure S5: Magnetic separation of the sorbents particles from the medium using a NdFeB magnet: a) particles in aqueous solution, b) after 30 seconds of magnetic separation and c) after 60 s of magnetic separation, Figure S6: Variation of SMX concentration on control experiments performed in absence of sorbent particles to assess the loss of SMX caused by other phenomena than adsorption on sorbents, Figure S7: Time profile of removal percentage of SMX at variable SMX initial concentration (40 and 80 mg/L) using Fe_3_O_4_@SiO_2_/SiTMC and Fe_3_O_4_@SiO_2_/SiCHIT, for 24h (1440 min), Figure S8: TGA curves of Fe_3_O_4_@SiO_2_/SiTMC and Fe_3_O_4_@SiO_2_/SiCHIT dried particles before and after SMX adsorption tests (C_0_ = 80 mg/L, contact time 8h), Table S1: BET surface area (S_BET_) and theoretical surface area estimated (S), Table S2: Elemental microanalysis of pristine chitosan and TMC, Table S3: Kinetic parameters estimated from pseudo 1st order and pseudo 2nd order models and evaluation of its fittings for an initial SMX concentration (C_0_) of 40 and 80 mg/L, for Fe_3_O_4_@SiO_2_/SiTMC particles, Table S4: Kinetic parameters estimated from pseudo 1st order and pseudo 2nd order models and evaluation of its fittings for an initial SMX concentration (C_0_) of 40 and 80 mg/L, for Fe_3_O_4_@SiO_2_/SiCHIT particles, Table S5: Elemental microanalysis of Fe_3_O_4_@SiO_2_/SiTMC and Fe_3_O_4_@SiO_2_/SiCHIT dried particles before and after SMX adsorption tests (C_0_ = 80 mg/L, contact time 8h).
