# Supplementary material for: Trimethyl Chitosan/Siloxane-Hybrid Coated Fe3O4 Nanoparticles for the Uptake of Sulfamethoxazole from Water
Source: Molecules. 2019 May 21;24(10):1958. doi: 10.3390/molecules24101958 (PMC6572444; doi:10.3390/molecules24101958)
Supplement: Supplementary file 1 [file molecules-24-01958-s001.pdf]

# Supporting Information

# Trimethyl Chitosan/Siloxane-Hybrid Coated $Fe_3O_4$ Nanoparticles for the Uptake of Sulfamethoxazole from Water

**Sofia F. Soares, Tiago Fernandes, Tito Trindade and Ana L. Daniel-da-Silva ***

[1] CICECO-Aveiro Institute of Materials, Department of Chemistry, University of Aveiro, 3810-193 Aveiro, Portugal; sofiafsoares@ua.pt (S.F.S.); jtfernandes@ua.pt (T.F.); tito@ua.pt (T.T.)

* Correspondence: ana.luisa@ua.pt; Tel.: +351-234-370-368

## A. Quantification of sulfamethoxazole in aqueous solutions

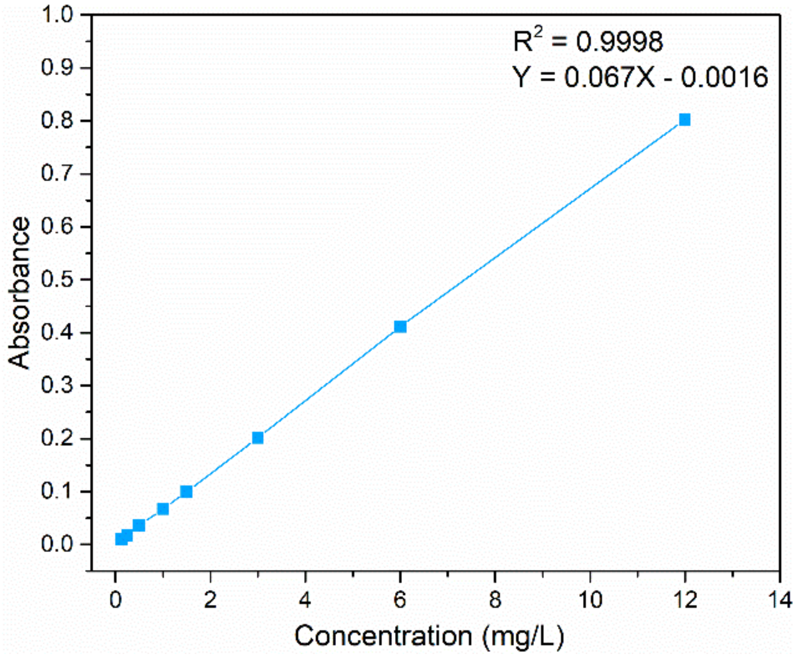


**Figure S1.** Calibration curve for sulfamethoxazole determination using UV-Vis spectroscopy.

**B. Characterization chitosan and derivatives**

The quaternization degree (QD) of the quaternary chitosan was calculated from the $^{1}H$ NMR spectra by using the Equation (S1),

$$DQ = \frac{I_{CH3}/9}{I_{H2-6}/6} \times 100 \qquad \textbf{(S1)}$$

where DQ (%) is the quaternization degree as a percentage, $I_{CH3}$ is the integral of the trimethylated amine peak, and $I_{H2\text{-}6}$ is the integral of the chitosan polymer chain.

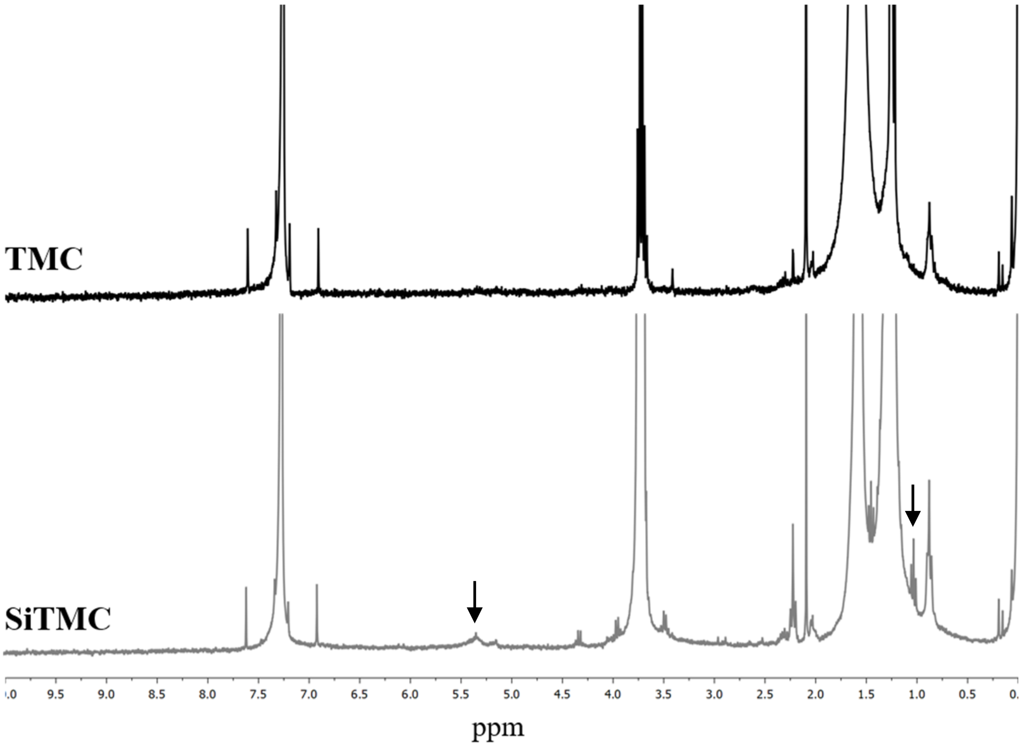


**Figure S2**. $^{1}H$ NMR spectra of TMC and SiTMC (in $CDCl_3$).

## C. Characterization of nanoparticles

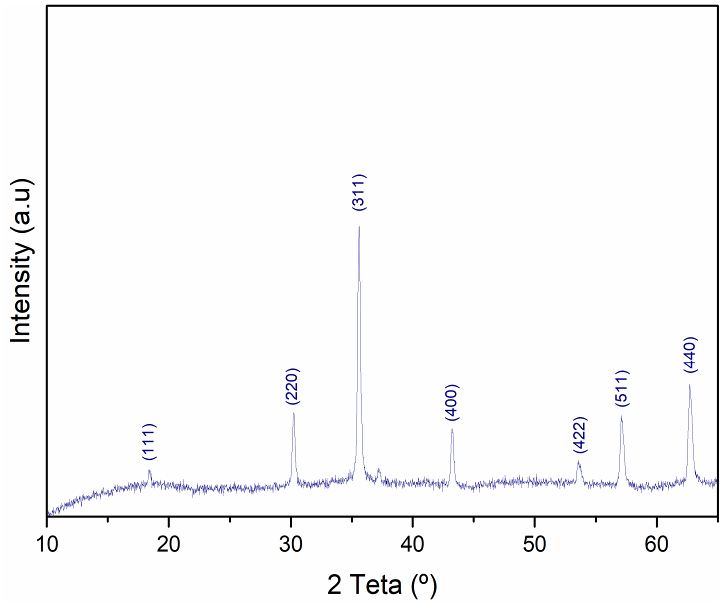


**Figure S3**. Powder XRD pattern of magnetite.

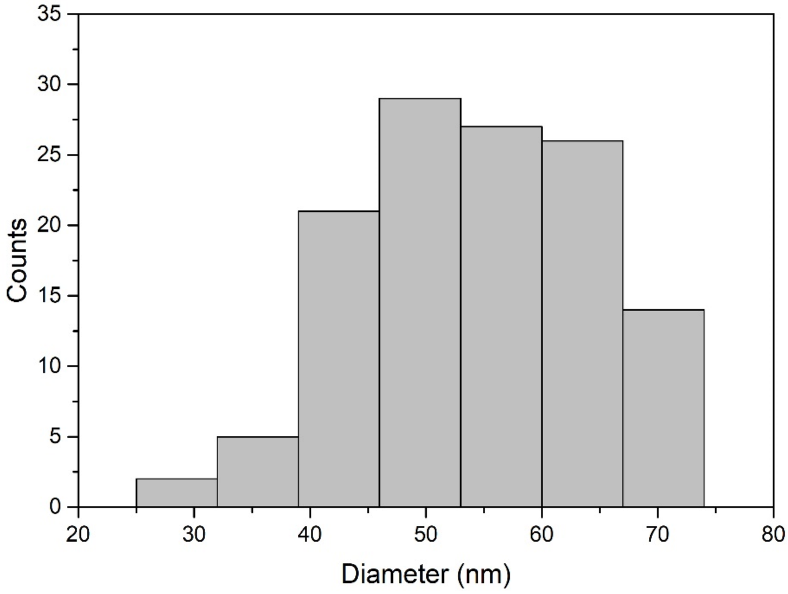


**Figure S4**. Histogram of particle diameter of $Fe_3O_4$ nanoparticles.

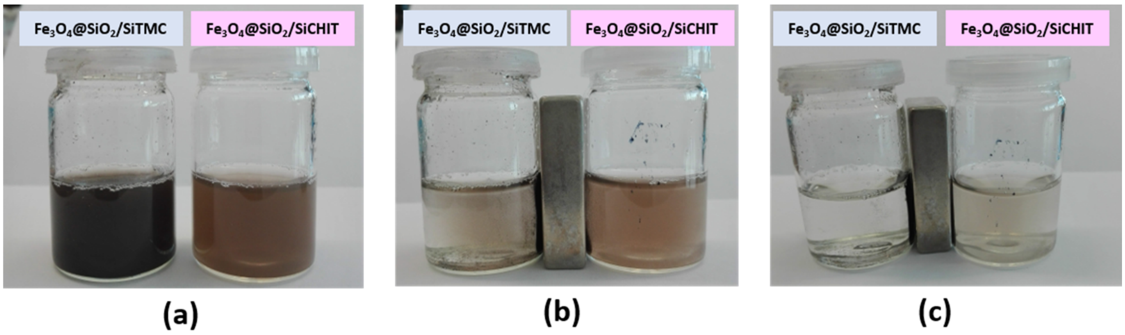


**Figure S5.** Magnetic separation of the sorbents particles from the medium using a NdFeB magnet: a) particles in aqueous solution, b) after 30 seconds of magnetic separation and c) after 60 seconds of magnetic separation.

**Table S1.** BET surface area ($S_{BET}$) and theoretical surface area estimated (S).

| Sample | $S_{BET}$ ($m^2/g$) | S ($m^2/g$) |
|---|---|---|
| $Fe_3O_4$ | 13.6 | 21.4 |
| $Fe_3O_4$@$SiO_2$/SiTMC | 7.03 | 22.7 |
| $Fe_3O_4$@$SiO_2$/SiCHIT | 7.74 | 23.8 |

The theoretical surface area was calculated using Equation (S2),

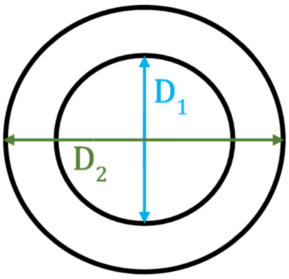


$$S = \frac{6D_2^2}{\rho_2(D_2^3 - D_1^3) + \rho_1 D_1^3} \qquad \textbf{(S2)}$$

where $D_1$ and $\rho_1$ is the diameter and density of core particle (magnetite), respectively, $D_2$ is the total diameter of the coated particle, and $\rho_2$ is the density of shell materials that was assumed to be identical to amorphous silica.

**Table S2.** Elemental microanalysis of pristine chitosan and TMC.

| Sample | C (%) | H (%) | N (%) |
|---|---|---|---|
| TMC | 36.8 | 6.3 | 6.1 |
| Chitosan | 39.7 | 6.5 | 7.3 |

**D. Uptake of sulfamethoxazole**

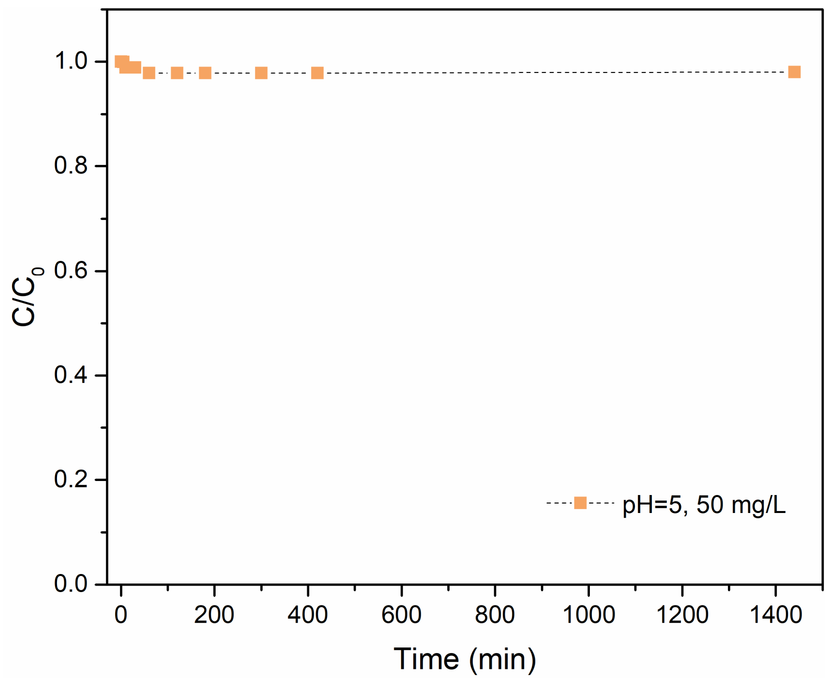


**Figure S6.** Variation of SMX concentration on control experiments performed in absence of sorbent particles to assess the loss of SMX caused by other phenomena than adsorption on sorbents.

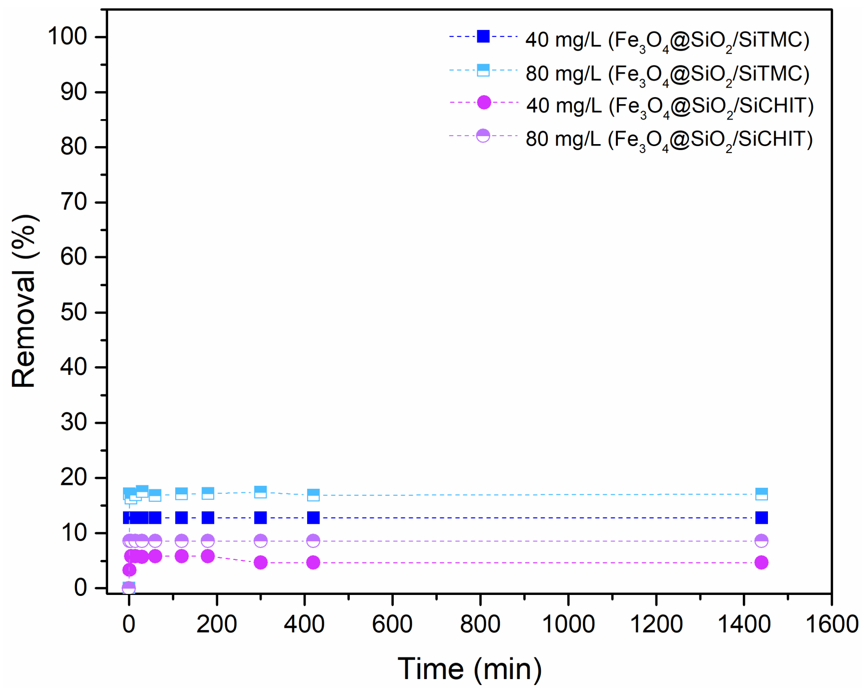


**Figure S7**. Time profile of removal percentage of SMX at variable SMX initial concentration (40 and 80 mg/L) using $Fe_3O_4$@$SiO_2$/SiTMC and $Fe_3O_4$@$SiO_2$/SiCHIT, for 24h (1440 min).

**E. Kinetics modeling and Goodness of the Fittings**

The kinetic models of pseudo-first and pseudo-second order were used to analyze the kinetic data. These two models assume that the adsorption is the rate-limiting step, rather than the diffusion of solute molecules. The kinetic equations are detailed below:

The pseudo-first kinetic model is given by the equation (S3), where $k_1$ ($min^{-1}$) is the pseudo-first order rate constant (Lagergren, 1907).

$$q_t = q_e\left(1 - e^{-k_1 t}\right) \qquad \textbf{(S3)}$$

The pseudo-second kinetic model is given by the equation (S4), where $k_2$ ($g.mg^{-1}.min^{-1}$) is the pseudo-second order rate constant (Ho and McKay, 1999).

$$q_t = \frac{k_2 q_e^2 t}{1 + k_2 q_e t} \qquad \textbf{(S4)}$$

The goodness of the fittings was evaluated by calculating the coefficient of determination ($R^2$) and Chi-square test value ($\chi^2$), expressed by equations S5 and S6, respectively,

$$R^2 = 1 - \frac{\sum_{i=1}^{n}(y_i - \hat{y}_i)^2}{\sum_{i=1}^{n}(y_i - \bar{y})^2} \qquad \textbf{(S5)}$$

$$\chi^2 = \sum_{i=1}^{n} \frac{(y_i - \hat{y}_i)^2}{\hat{y}_i} \qquad \textbf{(S6)}$$

where $y_i$ and $\hat{y}_i$ are the experimental and model predicted values respectively, $\bar{y}$ is the mean of the experimental data and $n$ is the sample size.

The model parameters and goodness of the fittings are depicted in Table S3 and S4.

**Table S3.** Kinetic parameters estimated from pseudo 1st order and pseudo 2nd order models and evaluation of its fittings for an initial SMX concentration ($C_0$) of 40 and 80 mg/L, for $Fe_3O_4$@$SiO_2$/SiTMC particles.

| | **Pseudo 1st order** | | | **Pseudo 2nd order** | | | |
|---|---|---|---|---|---|---|---|
| **$C_0$ (mg/L)** | $R^2$ ($\chi^2$) | $k_1$ ($min^{-1}$) | $q_e$ (mg. $g^{-1}$) | $R^2$ ($\chi^2$) | $k_2$ ($g.mg^{-1}.min^{-1}$) | $q_e$ (mg. $g^{-1}$) | $q_e$ exp ($mg.g^{-1}$) |
| **40** | 0.9813 (0.1875) | 2.0903 | 10.97 | 0.9883 (0.1441) | 0.4973 | 11.07 | 11.14 |
| **80** | 0.9968 (0.0786) | 3.2478 | 26.92 | 0.9975 (0.0601) | 0.7209 | 26.99 | 27.55 |

**Table S4.** Kinetic parameters estimated from pseudo 1st order and pseudo 2nd order models and evaluation of its fittings for an initial SMX concentration ($C_0$) of 40 and 80 mg/L, for $Fe_3O_4$@$SiO_2$/SiCHIT particles.

| | **Pseudo 1st order** | | | **Pseudo 2nd order** | | | |
|---|---|---|---|---|---|---|---|
| **$C_0$ (mg/L)** | $R^2$ ($\chi^2$) | $k_1$ ($min^{-1}$) | $q_e$ (mg. $g^{-1}$) | $R^2$ ($\chi^2$) | $k_2$ ($g.mg^{-1}.min^{-1}$) | $q_e$ (mg. $g^{-1}$) | $q_e$ exp ($mg.g^{-1}$) |
| **40** | 0.9998 (0.0008) | 0.8527 | 4.42 | 0.9862 (0.0705) | 0.3428 | 4.49 | 3.65 |
| **80** | 0.9934 (0.0809) | 1.9722 | 13.51 | 0.9974 (0.04323) | 0.3971 | 13.61 | 13.63 |

**F. Equilibrium Isotherm Modeling and Goodness of the Fittings**

Adsorption isotherms were analyzed according to the nonlinear form of Langmuir, Freundlich and Sips isotherms. The adequacy of each model and the respective equation is presented below:

***Langmuir isotherm:*** The Langmuir sorption isotherm applies to systems where the adsorption of the solute occurs as a monolayer on a surface containing a finite number of identical sites. The adsorption must be uniform with no transmigration of adsorbate in the surface plane [1,2].The non-linear form of the Langmuir model is given by the following equation (S7):

$$q_e = \frac{q_L K_L C_e}{1+K_L C_e} \qquad \textbf{(S7)}$$

where, $q_L$ (mg/g) is the monolayer adsorption capacity per unit of adsorbent and $K_L$ (L/mg) is the Langmuir adsorption constant related to the affinity of binding sites.

***Freundlich isotherm:*** The Freundlich isotherm assumes that the adsorption occurs on a heterogeneous surface, resulting in adsorption sites of varying energy. Since this model is based on an exponential equation, it assumes that the concentration of adsorbate on the adsorbent surface increases with the increase of adsorbate concentration. In general terms, this model assumes that the adsorption can occur based on multiple layers [1,3]. The non-linear form of the Langmuir models is described based on the following equation (S8):

$$q_e = k_F {C_e}^{1/n} \qquad \textbf{(S8)}$$

where $k_F$ ($mg^{(1-1/n)}.L^{(1/n)}.g^{-1}$) is the Freundlich constant and 1/n is the heterogeneity factor which varies between 0 and 1.

***Sips isotherm:*** The Sips isotherm model, also known as the Langmuir- Freundlich isotherm, combines the characteristics of both Langmuir and Freundlich isotherms [1,4]. At high solute concentrations this model predicts the monolayer adsorption capacity as in the Langmuir model, whereas at low solute

concentrations it reduces to Freundlich model. The non-linear form of the Sips model is given by equation (S9):

$$q_e = \frac{q_s k_S C_e^{1/\beta_S}}{1+k_S C_e^{1/\beta_S}} \quad \textbf{(S9)}$$

where Ks $(mg/L/)^{-1/\beta_S}$ is the Sips isotherm model constant and $\beta_S$ ($0<\beta_S<1$) is the Sips isotherm model exponent.

## G. Robustness of sorbent particles

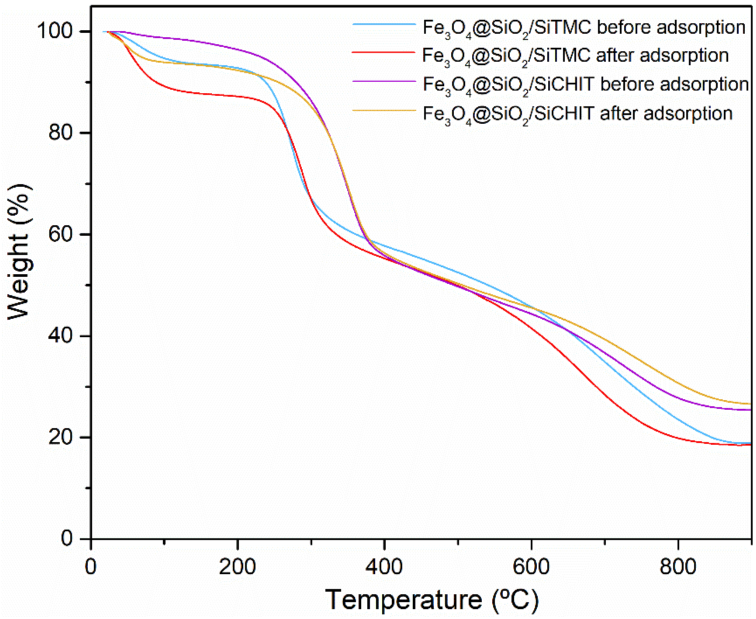


**Figure S8.** TGA curves of $Fe_3O_4@SiO_2$/SiTMC and $Fe_3O_4@SiO_2$/SiCHIT dried particles before and after SMX adsorption tests ($C_0$=80 mg/L, contact time 8h).

**Table S5.** Elemental microanalysis of $Fe_3O_4@SiO_2$/SiTMC and $Fe_3O_4@SiO_2$/SiCHIT dried particles before and after SMX adsorption tests ($C_0$=80 mg/L, contact time 8h).

| Sample | C (%) | H (%) | N (%) |
|---|---|---|---|
| $Fe_3O_4@SiO_2$/SiTMC before adsorption | 28.6 | 5.3 | 5.1 |
| $Fe_3O_4@SiO_2$/SiTMC after adsorption | 31.6 | 5.1 | 5.8 |
| $Fe_3O_4@SiO_2$/SiCHIT before adsorption | 28.5 | 5.8 | 5.3 |
| $Fe_3O_4@SiO_2$/SiCHIT after adsorption | 33.8 | 5.3 | 5.6 |
